# Potential role of postbiotics in supporting lean mass preservation and weight-loss sustainability during GLP-1–based anti-obesity therapy

**DOI:** 10.3389/fnut.2026.1794882

**Published:** 2026-03-24

**Authors:** Emel Cengiz Kaynakcı

**Affiliations:** 1 Department of Medical Biotechnology, Institute of Health Sciences, Akdeniz University, Antalya, Türkiye; 2 Department of Nutrition and Dietetics, Faculty of Health Sciences, Akdeniz University, Antalya, Türkiye; 3 Faculty of Health Sciences Research Laboratory, Akdeniz University, Antalya, Türkiye

**Keywords:** body composition, GLP-1 receptor agonists, gut–brain axis, lean mass preservation, postbiotics, resistance training, short-chain fatty acids, weight regain

## Abstract

Glucagon-like peptide-1 receptor agonists (GLP-1RAs) have reshaped obesity management by producing clinically meaningful weight loss, primarily through appetite suppression and reduced energy intake. However, rapid pharmacologically induced weight loss may be accompanied by unfavorable body-composition changes, including reductions in lean mass, and weight regain is common following treatment discontinuation, underscoring a major durability gap. Although preclinical studies link GLP-1 signaling to brown adipose tissue (BAT) activation and adipose tissue browning, human data indicate that BAT mass and thermogenic capacity in adulthood—particularly in obesity—are limited, constraining the contribution of energy expenditure to sustained weight loss. Beyond pharmacotherapy, emerging evidence suggests that gut microbiota–derived metabolites and postbiotics, such as short-chain fatty acids (SCFAs) and tryptophan-derived indoles, can modulate enteroendocrine function, inflammatory tone, insulin sensitivity, and gut–brain communication. While postbiotics are unlikely to replicate the magnitude of weight loss achieved with GLP-1RAs, their continuous, physiology-aligned mode of action positions them as biologically plausible adjuncts that may support weight-loss sustainability and improve the metabolic context for lean mass preservation. This mini-review integrates pharmacological, physiological, and nutritional perspectives to examine the mechanisms underlying GLP-1–induced weight loss and the physiological limits of thermogenic pathways in humans. It further discusses the potential complementary role of postbiotics within convergence-based strategies aimed at preserving lean mass and enhancing long-term obesity management.

## Introduction

Obesity is a chronic, relapsing disease driven by complex interactions among genetic susceptibility, neuroendocrine regulation, metabolic adaptation, and environmental exposure. Rather than reflecting a simple imbalance between energy intake and expenditure, obesity represents an actively defended physiological state in which compensatory mechanisms promote weight regain following weight loss attempts (1). This biological resistance has historically limited the long-term efficacy of lifestyle-based interventions and has motivated the development of pharmacological strategies targeting appetite and energy homeostasis.

Among these strategies, glucagon-like peptide-1 receptor agonists (GLP-1RAs) have emerged as the most effective anti-obesity pharmacotherapies to date. Initially developed for type 2 diabetes, GLP-1RAs have demonstrated robust weight loss in randomized controlled trials and real-world settings, leading to their repositioning as cornerstone obesity treatments (2, 3). Their dominant mechanism involves suppression of appetite and caloric intake via delayed gastric emptying and central satiety signaling mediated through vagal afferents and hypothalamic circuits (4, 5).

Human metabolic studies consistently show that reductions in energy intake account for most of the weight loss observed during GLP-1RA therapy, whereas increases in resting energy expenditure are modest or absent (3, 6). In contrast, preclinical data suggest that GLP-1 signaling can stimulate sympathetic outflow to adipose tissue, activate brown adipose tissue (BAT), and promote browning of white adipose depots (7, 8). However, translational studies in humans indicate that BAT mass and activity decline with age and obesity, limiting the quantitative contribution of thermogenesis to whole-body energy expenditure (9, 10).

Beyond total weight loss, the quality of weight loss has become an increasingly important clinical concern. Rapid pharmacologically induced weight reduction may be accompanied by losses of lean mass, particularly when energy deficits are large, protein intake is inadequate, and resistance exercise is not implemented. Lean mass supports resting metabolic rate, physical function, and long-term weight maintenance; thus, its loss may exacerbate metabolic adaptation and increase vulnerability to rebound weight gain following treatment discontinuation. Consequently, strategies that preserve lean mass during active GLP-1RA therapy and stabilize metabolic regulation during transition or withdrawal are essential components of sustainable obesity care.

In parallel, increasing attention has focused on the gut microbiota as a regulator of host metabolism and enteroendocrine signaling. Microbiota-derived metabolites, including short-chain fatty acids (SCFAs) and tryptophan-derived indoles, can modulate GLP-1 secretion, gut–brain communication, insulin sensitivity, and inflammatory tone (11–13). In this context, postbiotics—defined as non-viable microbial components or metabolites conferring health benefits—have emerged as promising nutritional tools capable of influencing metabolic homeostasis without the safety concerns associated with live microorganisms (14, 15). In this review, the term “postbiotic-rich dietary patterns” refers to dietary approaches that enhance endogenous production of microbiota- derived metabolites, particularly short-chain fatty acids, primarily through increased intake of fermentable dietary fibers (14, 16).

This mini-review critically examines the mechanisms underlying GLP-1–induced weight loss, the physiological limits of energy expenditure in humans, and the emerging evidence supporting postbiotics as complementary modulators within convergence-based strategies aimed at lean mass preservation and weight-loss sustainability.

## Central and peripheral control of energy balance: pharmacological GLP-1 signaling vs. microbiota-derived modulation

The regulation of energy balance reflects coordinated interactions between peripheral nutrient sensing and central neural integration. GLP-1RAs exert their anti-obesity effects primarily by amplifying signaling within this gut–brain axis, delivering a sustained satiety signal that overrides endogenous regulatory constraints (3, 5). Long-acting GLP-1RAs resist enzymatic degradation, resulting in prolonged receptor activation that far exceeds the magnitude and duration of physiological GLP-1 exposure (17).

Physiological GLP-1 does not function as a classical circulating hormone; it acts mainly through paracrine and neural mechanisms and is rapidly degraded (5). Pharmacological GLP-1RAs therefore represent an intentional exaggeration of a normally transient biological signal rather than a direct mimic of endogenous GLP-1 function (3, 18). This distinction explains both their clinical potency and the rapid attenuation of effects following treatment withdrawal.

Postbiotics engage the same regulatory circuitry indirectly. SCFAs stimulate GLP-1 secretion from intestinal L cells via free fatty acid receptors (FFAR2/3), enhancing endogenous GLP-1 release in a nutrient-dependent manner (11). Tryptophan-derived microbial metabolites activate aryl hydrocarbon receptor pathways, influencing enteroendocrine differentiation and gut–brain communication (13). While the magnitude of GLP-1 release induced by postbiotics is modest compared with pharmacological agonists, their effects are continuous and physiologically integrated (14).

This contrast between signal intensity and biological context has important implications. GLP-1RAs excel at inducing short-term weight loss by imposing a powerful external satiety signal, whereas postbiotics may contribute to stabilizing the endogenous signaling environment that governs appetite regulation and metabolic adaptation (1, 19).

## Adipose tissue plasticity and thermogenic capacity: BAT, browning, and the physiological ceiling in humans

The discovery of metabolically active BAT in adult humans raised expectations that obesity therapies might meaningfully increase energy expenditure through thermogenesis (9, 30). In rodents, central GLP-1 receptor activation stimulates sympathetic outflow to adipose tissue, induces thermogenic gene programs, and promotes browning of white adipose depots (7, 8).

In humans, however, BAT mass is limited, heterogeneous, and reduced in obesity, and even maximal activation contributes only modestly to total energy expenditure (10, 31). Clinical studies of GLP-1RAs generally do not demonstrate sustained increases in resting energy expenditure, reinforcing the view that appetite suppression—not thermogenesis—is the dominant driver of weight loss (2, 6).

Importantly, the physiological ceiling on BAT-mediated thermogenesis implies that increasing energy expenditure is unlikely to offset the metabolic adaptation associated with rapid weight loss. This shifts clinical emphasis toward interventions with stronger evidence for preserving lean mass—such as adequate dietary protein and resistance training—while complementary biological modulators like postbiotics may improve insulin sensitivity and inflammatory tone, thereby supporting metabolic resilience rather than directly increasing thermogenesis.

## Lean mass preservation during GLP-1–based weight loss: why body composition matters

A key translational issue in GLP-1RA-based obesity therapy is that substantial weight loss does not automatically translate into favorable body-composition outcomes. When caloric restriction is pronounced, fat-free mass loss becomes more likely, particularly in older adults, individuals with low protein intake, or those not engaging in resistance exercise. Lean mass loss is clinically relevant because it reduces physical function and resting metabolic rate, potentially amplifying compensatory responses and increasing susceptibility to weight regain (20, 21).

GLP-1RAs are not designed to exert anabolic effects on skeletal muscle; their primary role is induction of a negative energy balance. Therefore, lean mass preservation should be conceptualized as a parallel treatment target achieved through nutritional and behavioral strategies rather than pharmacology alone. Adequate dietary protein and progressive resistance training remain the most evidence-supported approaches to maintain muscle protein synthesis during weight loss.

Postbiotics are unlikely to prevent lean mass loss through direct anabolic mechanisms. Instead, their plausible contribution is indirect: by improving insulin sensitivity, reducing chronic low-grade inflammation, and supporting metabolic flexibility, postbiotics may enhance the efficiency of nutrient utilization and the anabolic response to protein and exercise. This positions postbiotics as context modulators that complement established lean mass–preserving interventions rather than substitutes for them (14, 15).

Although postbiotics are not expected to exert direct anabolic effects on skeletal muscle, their potential relevance to lean mass preservation lies in indirect metabolic pathways. Improvements in insulin sensitivity and reductions in chronic low-grade inflammation may enhance the anabolic responsiveness of skeletal muscle to dietary protein and resistance exercise. In addition, stabilization of gut–brain signaling and substrate utilization may reduce metabolic stress during sustained energy deficit, thereby supporting a physiological environment more permissive to lean tissue retention. These effects should be interpreted as context-supportive rather than causative, reinforcing the role of postbiotics as adjunctive modulators within comprehensive body-composition–focused weight-loss strategies (14, 16, 20, 21).

Importantly, there are currently no direct human interventional studies demonstrating that postbiotic supplementation preserves skeletal muscle mass during GLP-1–based obesity therapy. The proposed relevance of postbiotics for lean mass preservation remains mechanistic and indirect, largely extrapolated from evidence on insulin sensitivity, inflammatory modulation, and metabolic regulation. Accordingly, this hypothesis should be interpreted as biologically plausible but not yet supported by controlled human outcome trials specifically assessing body composition endpoints.

## Durability of weight loss and metabolic adaptation: what happens when the signal is withdrawn?

Weight regain following GLP-1RA discontinuation is common and often rapid, indicating that pharmacological appetite suppression does not permanently reset biological weight-defense systems (22, 23). Upon withdrawal, appetite rebounds, caloric intake increases, and body weight trends toward pre-treatment levels, consistent with classical models of adaptive thermogenesis and metabolic compensation (24, 25).

Postbiotics introduce a contrasting paradigm. Rather than imposing an external signal, they continuously shape the metabolic environment through interactions among diet, microbiota, and host physiology. SCFAs and related metabolites have been shown to influence insulin sensitivity, substrate utilization, and inflammatory tone in ways that persist beyond acute exposure (16, 26). Although postbiotics alone produce modest weight changes, their continuous mode of action suggests potential value in attenuating rebound responses during maintenance phases.

## Translational perspective: why GLP-1 and postbiotics should converge rather than compete

**Figure 1:**
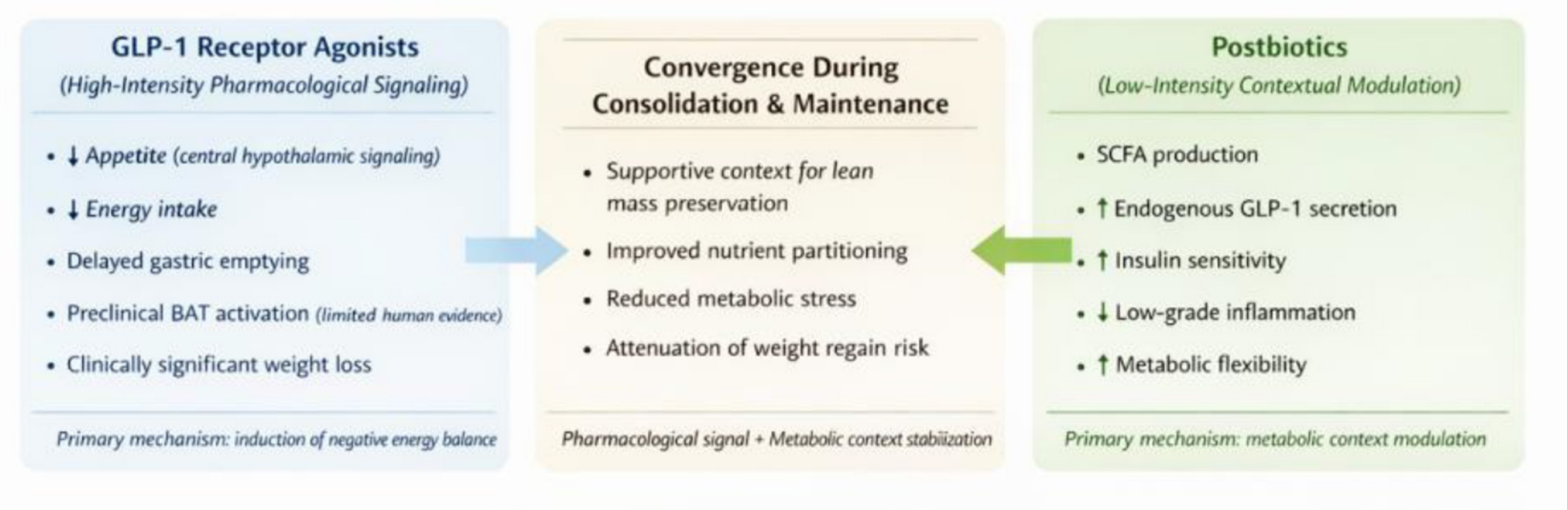
Conceptual convergence between GLP-1 receptor agonists and postbiotics in obesity management. GLP-1 receptor agonists induce weight loss primarily through appetite suppression and reduced energy intake via central and peripheral pathways. In contrast, postbiotics modulate metabolic regulation indirectly through *microbiota-derived signaling*, including *short-chain fatty acid production*, insulin *sensitivity, and inflammatory tone*. During consolidation and maintenance phases, these distinct mechanisms may converge to *support metabolic stability and lean mass preservation* within *comprehensive obesity treatment* strategies.

From a translational standpoint, GLP-1RAs and postbiotics operate at different hierarchical levels of metabolic control. GLP-1RAs deliver high-intensity, pharmacologically sustained signals that reliably induce weight loss, whereas postbiotics provide low-intensity but continuous modulation of gut-derived signaling that shapes metabolic context (3, 14). A comparative overview of these complementary mechanisms is summarized in Table 1 and conceptually illustrated in Figure 1.

**Table 1:** Evidence-based comparison of GLP-1 receptor agonists and postbiotics in obesity management.

**Domain**	**GLP-1 receptor agonists (GLP-1RAs)**	**Postbiotics**	**Key citations**
Primary role	Pharmacological induction of weight loss through sustained appetite suppression and central satiety signaling	Nutritional modulation of metabolic and inflammatory context; plausible adjunct during consolidation/maintenance	(3, 14, 18, 27)
Magnitude of weight loss	Large and clinically meaningful weight loss in human RCTs (e.g., semaglutide 2.4 mg/week associated with ~10%−15% mean weight loss over 68 weeks in the STEP program)	Modest and heterogeneous effects in human RCTs (typically 4–12-week interventions); stronger anti-obesity signals reported in high-fat diet animal models	(22, 27–29)
Effect on energy intake	Marked reduction in energy intake via gut–brain axis mechanisms	Indirect modulation via SCFA and enteroendocrine pathways; not a “high-intensity” satiety signal	(4, 11, 16)
Thermogenesis and BAT	Rodent BAT/browning effects reported; limited quantitative contribution in adult humans	BAT/browning effects reported predominantly in high-fat diet animal studies; human translation constrained	(7, 10, 28, 29)
Lean mass implications	Lean mass loss can accompany rapid weight reduction unless mitigated by protein and resistance training	No direct anabolic effect; may support anabolic responsiveness indirectly via insulin sensitivity/inflammation	(16, 20, 21, 27)
Durability after discontinuation	Weight regain commonly observed after cessation	Effects depend on continued intake/dietary pattern; proposed supportive role in maintenance	(22, 23, 27)
Overall strength of evidence	Strong: large-scale human RCTs and long-term extensions	Emerging: mechanistic + preclinical + limited human intervention evidence	(18, 27–29)
Clinical positioning (this review)	Weight-loss induction phase	Adjunctive metabolic-context support during consolidation/maintenance (especially post-therapy)	(14, 27)

Evidence supporting postbiotics in obesity management is derived predominantly from mechanistic and preclinical studies, with an emerging but heterogeneous body of human trial evidence indicating modest and variable effects on anthropometric outcomes. Accordingly, postbiotics should be interpreted as adjunctive nutritional strategies rather than pharmacological alternatives to GLP-1 receptor agonists, with the most plausible utility during consolidation and maintenance phases aimed at improving metabolic context and supporting long-term weight-loss sustainability (14, 27–29).

Importantly, accumulating preclinical evidence indicates that certain postbiotic preparations can attenuate weight gain, reduce adiposity, and activate thermogenic pathways, including brown adipose tissue signaling, particularly in high-fat diet–fed rodent models. These effects have been attributed to improvements in insulin sensitivity, modulation of lipid metabolism, and activation of enteroendocrine and thermogenic gene programs. However, translation of these findings to adult human obesity remains constrained by fundamental physiological differences, including limited brown adipose tissue mass and reduced thermogenic capacity. In this context, postbiotics are more plausibly positioned not as primary weight-loss interventions, but as nutritional strategies that may be continued after GLP-1–based pharmacotherapy to support metabolic stability, mitigate weight regain, and preserve lean mass during the post-treatment phase (10, 14, 16, 22, 26).

From a clinical nutrition perspective, postbiotics should not be positioned as weight-loss agents, nor as substitutes for pharmacological GLP-1 receptor agonists. Rather, their most plausible role emerges during the consolidation and maintenance phases of GLP-1–based therapy, when preserving metabolic stability and lean tissue becomes a priority. In this context, postbiotic-rich dietary patterns or defined postbiotic preparations may support insulin sensitivity, attenuate low-grade inflammation, and stabilize enteroendocrine signaling, thereby creating a more favorable metabolic environment for protein utilization and resistance training–induced adaptations. Such an approach aligns with the concept of metabolic context modulation, in which nutritional interventions reinforce physiological resilience rather than impose additional caloric restriction (3, 12, 14, 16).

A practical convergence framework can be described in three phases. During the induction phase, GLP-1RAs create a robust energy deficit via appetite suppression. During the consolidation phase, clinical priorities expand to include lean mass preservation through adequate protein intake and resistance training, while postbiotic-informed strategies may support insulin sensitivity and inflammatory balance. During the maintenance phase, durability becomes paramount; persistent dietary patterns, physical activity, and metabolic-context modulation aim to attenuate rebound once pharmacological pressure is reduced.

## Conclusion

GLP-1 receptor agonists have fundamentally reshaped obesity treatment by enabling unprecedented pharmacological weight loss. However, their efficacy is driven primarily by appetite suppression rather than sustained increases in energy expenditure, and adult human physiology imposes a clear ceiling on thermogenic contributions. Rapid weight loss under GLP-1RA therapy may compromise lean mass and is frequently followed by weight regain upon treatment discontinuation. Postbiotics operate through a distinct, complementary mechanism: continuous modulation of gut-derived signaling pathways that influence enteroendocrine function, insulin sensitivity, inflammation, and metabolic adaptation. Although postbiotics cannot replicate the magnitude of GLP-1–induced weight loss, they may help stabilize the metabolic context in which weight loss is maintained and lean mass is preserved. It should be acknowledged that evidence supporting postbiotics in the context of GLP-1–based obesity therapy remains largely indirect and mechanistic, with limited data from well-controlled human intervention trials specifically addressing lean mass outcomes. Accordingly, the present mini-review is intended to provide a hypothesis-generating and translational framework rather than definitive clinical guidance. Future randomized studies integrating body-composition endpoints, dietary protein adequacy, resistance training, and defined postbiotic interventions will be essential to clarify the magnitude and clinical relevance of these interactions. Effective long-term obesity management is therefore unlikely to emerge from competition between pharmacological and nutritional approaches. Instead, convergence strategies that combine the potency of GLP-1RAs for weight-loss induction with interventions that support lean mass preservation and metabolic durability represent a more biologically coherent and clinically realistic pathway forward. Within this framework, defining evidence-based priorities for future research becomes critical to translate these complementary strategies into clinically meaningful outcomes.

## Take-home perspective

The central challenge in obesity management is no longer achieving weight loss, but maintaining it without sacrificing functional lean tissue. GLP-1 receptor agonists provide powerful induction tools, yet their effects are contingent on continuous administration and do not dismantle biological weight-defense systems. Postbiotics should not be judged by the standards applied to anti-obesity drugs; their value lies in long-term metabolic support rather than rapid weight reduction. A division of labor—pharmacology for initiation, nutrition and microbial-context modulation for preservation—offers a pragmatic and biologically defensible model for sustainable obesity care.

## Future directions and research priorities

Future research should prioritize well-controlled human intervention studies integrating GLP-1–based pharmacotherapy with defined postbiotic preparations, with explicit assessment of body composition, lean mass preservation, and post-discontinuation weight trajectories. Harmonization of postbiotic definitions, dosing strategies, and mechanistic endpoints will be essential to determine clinical relevance beyond preclinical models.
